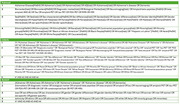# A Systematic Review of Disparities in Alzheimer's Diagnosis: The Overlooked Impact of Gender and Ethnoracial Diversity on Biomarker Expression

**DOI:** 10.1002/alz70856_105448

**Published:** 2026-01-08

**Authors:** Cynthia Isabel Smith, Olisaemeka Ogbue, Nasra Gathoni, Karen Blackmon, Tamlyn J Watermeyer, Jasmit Shah, Chinedu Udeh‐Momoh

**Affiliations:** ^1^ Brain and Mind Institute, Aga Khan University, Nairobi, Nairobi, Kenya; ^2^ Aga Khan University, Nairobi, Nairobi, Kenya; ^3^ Kings College London, London, London, United Kingdom; ^4^ Brain and Mind Institute, Aga Khan University, Nairobi, Kenya; ^5^ National Institute of Health Applied Research Collaboration North East & Cumbria, Newcastle‐upon‐Tyne, England, United Kingdom; ^6^ Edinburgh Dementia Prevention, Centre for Clinical Brain Sciences, College of Medicine and Veterinary Medicine, University of Edinburgh, Edinburgh, United Kingdom; ^7^ Female Brain Health and Endocrine Research (FemBER) Consortium, Newcastle, Edinburgh, London, United Kingdom; ^8^ University of Northumbria, Newcastle upon tyne, England, United Kingdom; ^9^ Global Brain Health Institute, University of California, San Francisco, NC, USA; ^10^ Wake Forest University, School of Medicine, Winston‐Salem, NC, USA

## Abstract

**Background:**

Biomarkers hold significant promise for the early diagnosis of dementias like Alzheimer's disease (AD). However, less than 5% of research data originates from Black African or African American populations, despite Africa's increasing dementia burden. Additionally, women are disproportionately affected by dementia globally, a trend that is expected to persist. This systematic review aims to synthesize studies on sex and gender differences in AD biomarkers, with a focus on African populations.

**Method:**

We have conducted systematic searches across PubMed, Scopus, Web of Science, SciELO, and African journals online for articles published between 2000 and 2024. Our focus is on neuroimaging, genetic, cerebrospinal fluid, and blood‐based biomarkers in African populations. Studies included explore AD and its biomarkers, particularly addressing sex, gender, and ethnoracial influences. The review adheres to PRISMA guidelines, is registered on PROPSERO CRD42024588618, and A meta‐analysis will be conducted to evaluate sex‐ and ethnoracial‐specific variations in AD biomarker levels across identified studies.

**Result:**

Preliminary findings from the literature reveal sex‐specific differences in AD biomarkers, with women are more likely to progress to dementia and be diagnosed with AD, even when biomarker levels are comparable with men. Importantly, African ethnicity has also been shown to influence AD biomarker expression, although research remains limited and inconsistent.

**Conclusion:**

There is an urgent need for research in African populations to improve our understanding of AD biomarkers, particularly regarding sex and gender differences. Our meta‐analysis will identify critical gaps in the literature. Furthermore, initiatives like FemBER‐Africa are pivotal for advancing AD and biomarker research on the continent. This review will enhance global understanding of AD biomarkers and provide targeted insights to advance dementia research in Africa.